# Hyaluronic acid modified covalent organic polymers for efficient targeted and oxygen-evolved phototherapy

**DOI:** 10.1186/s12951-020-00735-x

**Published:** 2021-01-06

**Authors:** Fangpeng Shu, Taowei Yang, Xuefeng Zhang, Wenbin Chen, Kaihui Wu, Junqi Luo, Xumin Zhou, Guochang Liu, Jianming Lu, Xiangming Mao

**Affiliations:** 1 Department of Urology, Guangzhou Women and Children’s Medical Center, Guangzhou Medical University, Guangzhou, China; 2 Department of Urology, Zhujiang Hospital of Southern Medical University, Guangzhou, China; 3 Department of Urology, First Affiliated Hospital of Soochow University, 899 Pinghai Road, Suzhou, 215031 China

**Keywords:** Targeted therapy, Photothermal therapy, Photodynamic therapy, Covalent organic polymers, Hypoxia tumor

## Abstract

The integration of multiple functions with organic polymers-based nanoagent holds great potential to potentiate its therapeutic efficacy, but still remains challenges. In the present study, we design and prepare an organic nanoagent with oxygen-evolved and targeted ability for improved phototherapeutic efficacy. The iron ions doped poly diaminopyridine (FeD) is prepared by oxidize polymerization and modified with hyaluronic acid (HA). The obtained FeDH appears uniform morphology and size. Its excellent colloidal stability and biocompatibility are demonstrated. Specifically, the FeDH exhibits catalase-like activity in the presence of hydrogen peroxide. After loading of photosensitizer indocyanine green (ICG), the ICG@FeDH not only demonstrates favorable photothermal effect, but also shows improved generation ability of reactive oxygen species (ROS) under near-infrared laser irradiation. Moreover, the targeted uptake of ICG@FeDH in tumor cells is directly observed. As consequence, the superior phototherapeutic efficacy of the targeted ICG@FeDH over non-targeted counterparts is also confirmed in vitro and in vivo. Hence, the results demonstrate that the developed nanoagent rationally integrates the targeted ability, oxygen-evolved capacity and combined therapy in one system, offering a new paradigm of polymer-based nanomedicine for tumor therapy.
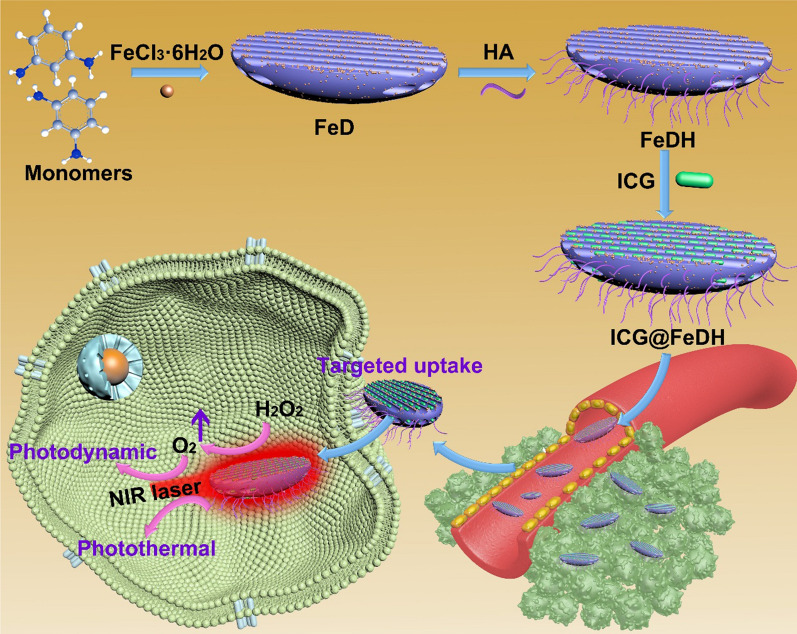

## Introduction

Nanomaterials-enabled therapy provides unprecedented opportunities to increase the therapeutic efficacy and specificity of tumor treatments in the past decades [1–3]. Nanocarriers-based delivery system can effectively improve the tumor accumulation of drugs and reduces the undesirable side effects on normal tissues based on the enhanced permeability and retention (EPR) effect [4]. More importantly, various functional nanomaterials have been applied to mediate new alternatives for tumor treatment with lower side effects [5, 6]. Phototherapy, including photothermal and photodynamic therapy, has drawn widespread interest in recent years due to its minimal invasion and controllable spatiotemporal selectivity [7, 8]. In many previous studies, carbon- [9–11], semiconductor- [12–14], and metal-based nanomaterials [15–18] have been developed as photothermal agents for photothermal therapy of tumor. However, those inorganic nanomaterials are always debated with their biodegradability and long-term in vivo retention [19]. With regard to photodynamic therapy, the photosensitizers as small molecules are required to be loaded into nanocarriers in order to prevent the photoblench process and improve the pharmacokinetics [20]. Therefore, the development of new type of nanocarriers is still urgently needed.

Covalent organic polymers, which are prepared by cross-linking organic molecules via covalent bonds, possess preferable stability under physiological conditions than traditional polymeric micelles and vesicles [21]. In several previous studies, covalent organic polymers have been demonstrated for chemo and phototherapy [22, 23]. However, as a newly emerged type of nanocarriers, covalent organic polymers still needs to be rationally functionalized for improved therapeutic efficacy. Liu’s group demonstrated nanoscale covalent organic polymers as a biodegradable nanomedicine for combined chemotherapy and photodynamic therapy [24, 25]. Porphyrinic covalent organic polymers have also been prepared for photothermal and photodynamic therapy [26]. Despite the excellent phototherapeutic efficacy, some drawbacks should be conquered in the design of covalent organic polymer-based nanomedicine. For instance, the hypoxia condition in tumor microenvironment severely hinders the efficacy of oxygen-dependent photodynamic therapy and even causes drug resistance or tumor metastasis [27, 28]. Nanoenzyme, who can mimic the activity of natural catalase, has been widely integrated with nanoagents for oxygen-evolving therapy by catalyzing the decomposition of hydrogen peroxide in tumor microenvironment [29, 30]. Therefore, the fabrication of covalent organic polymers with catalase-like activity is worthwhile to realize more efficient phototherapy.

In this work, a covalent organic polymer-based nanoagent is developed for targeted and oxygen-evolving phototherapy of tumor. Specifically, nano-scaled polydiaminopyridine nanoparticles doped with iron ions (FeD) is prepared and conjugated with hyaluronic acid (HA), obtaining the designed nanoagent FeDH. With the loading of a photosensitive molecule indocyanine green (ICG), the ICG@FeDH holds potential to combine several advantages based on following considerations: first, the FeDH is constructed from organic molecules by strong covalent bonds, ensuring the excellent biocompatibility and colloidal stability under physiological condition; second, the iron ions can be doped into the nanoagent and render it with catalase-like activity, thereby overcoming the tumor hypoxia to improve the phototherapeutic efficacy. The iron ions might also facilitate the clearance of the developed nanoagent in comparison to other commonly used inorganic nanoenzyme, such as platinum [31], manganese dioxide [32] and other metal oxides [33, 34]. Last, the modification of HA endows FeDH with targeted ability towards tumor cells. Combining the catalase activity, photothermal and photodynamic effect of loaded ICG as well as the targeted ability of HA, the ICG@FeDH can realize more efficient phototherapeutic efficacy under near-infrared (NIR) laser irradiation.

## Materials and methods

### Materials

2,6-diaminopyridine, FeCl_3_·6H_2_O and Sodium hyaluronate were commercially provided by Shanghai Aladdin Reagent CO, Ltd. (China). *N*-hydroxysuccinimide (NHS) and 1-(3-Dimethylaminopropyl)-3-ethylcarbodiimide hydrochloride (EDC·HCl) were brought from Sigma-Aldrich Trading Co., Ltd. (China). [Ru(dpp)_3_]Cl_2_ (RDPP), 1,3-diphenylisobenzofuran (DPBF) and 2′,7′-dichlorofluorescein diacetate (DCFH-DA)were purchased from Shanghai Medpep Co., Ltd (China). Roswell Park Memorial Institute (RPMI) 1640, fetal bovine serum (FBS), penicillin–streptomycin, and trypsin were supplied by GIBCO Invitrogen Corp. (USA). Cell counting kit-8 (CCK-8) and 4′,6-Diamidino-2-phenylindole (DAPI) were provided by Shanghai Yeasen Biotech Co., Ltd. (China). All other reagents were received and used without further purification. Deionized (DI) water was obtained from experimental water purification system.

### Synthesis of FeD nanoparticles

In a 50 mL round-bottom flask, 8.0 mmol of FeCl_3_•6H_2_O was stirred in 20 mL of deionized water for 0.5 h. Then, 2.0 mmol of 2, 6-diaminopyridine was added and the mixture was heated at 40 °C for 24 h. The obtained FeD nanoparticles were collected by dialysis.

### Preparation of FeDH

First, 0.1 g of HA, 36.2 mg of EDC and 27.9 mg of NHS were stirred in 10 mL of deionized water for 4 h. Then, 10 mL of FeD solutions (5 mg/mL) was added and reacted with activated HA for another 20 h. The HA modified FeD (denoted as FeDH) were obtained by high-speed centrifugation. To obtain ICG-loaded FeDH (ICG@FeDH), 5 mg of ICG was added in FeD solutions and the modification is proceeded with the same steps. The samples were isolated and purified by centrifugation.

### In vitro photothermal effect

To evaluate the photothermal performance of ICG@FeDH (or ICG@FeD), 0.5 mL of ICG@FeDH (or ICG@FeD) solution at different concentrations (100, 50, 25 μg/mL) was added into a Eppendorf tube (0.5 mL) and irradiated by the 808 nm laser (1 W/cm^2^, 5 min). The temperature changes were timely measured by an infrared thermal imaging camera. As controls, the temperature of pure water or FeDH (or ICG@FeD) solution with concentration of 100 μg/mL were also recorded under the same conditions. The photostability of ICG@FeDH (or ICG@FeD) was also assessed by heating and natural cooling rounds of 808 nm laser (1 W/cm^2^, 5 min).

### Assessment of oxygen generation ability

5 mL of H_2_O_2_ (2 mM) was mixed with 5 mL of FeDH dispersion with a dissolved oxygen meter inserted in the solution. The pure water and H_2_O_2_ solutions were also tested under the same conditions for comparison.

To evaluate the intracellular oxygen-evolving ability of FeDH, an oxygen-sensitive probe molecule RDPP was used to monitor the intracellular oxygen level. In brief, cells were seeded in culture dishes and then installed with RDPP (10 µM) at 37 °C for 2 h. Subsequently, the cells were treated with FeDH at 200 µg/mL for another 2 h. Finally, the human prostatic cancer cells (PC-3 cells) were rinsed with PBS for several times and directly imaged by confocal laser scanning microscope under an excitation of 488 nm.

### Reactive oxygen species detection

Typically, DPBF was used as a probe molecule to detect the singlet oxygen generation of different samples [35]. 1 mL of ICG@FeDH (or ICG@FeD) aqueous solution at concentration of 100 μg/mL was mixed with 50 μL of DPBF dissolved in dimethyl sulfoxide (10 mM) and irradiated by a 808 nm laser (1 mW/cm^2^). The absorbance at 419 nm was measured by a UV–vis spectrophotometer at different time intervals. In addition, singlet oxygen sensor green (SOSG) was also used to evaluate the singlet oxygen generation efficiency of ICG@FeDH and ICG@FeD. Typically, 5 µL of SOSG stock solution (5 mM) was added into 1 mL of ICG@FeDH (or ICG@FeD) solution (0.1 mg mL^−1^). The solution was irradiated by a 808 nm laser (1 W/cm^2^) for various times or kept in the dark as the control, and fluorescence intensity of SOSG at 525 nm was measured to determine the amount of singlet oxygen.

To investigate the intracellular ROS generation, PC-3 cells were seeded in culture dishes and incubated with ICG@FeDH (50 μg/mL) for 4 h. Then the cells were incubated with DCFH-DA (2 μM) for 0.5 h. After removing the excess probe, the cells were further illuminated by 808 nm laser for 10 min, and imaged by CLSM under the excitation of 808 nm. The cells treated with complete medium alone, laser irradiation alone, and ICG@FeDH alone were also stained with DCFH-DA for control. Moreover, the cells pre-treated with HA were also set as the receptor-inhibition group, in which the same protocol was performed except that the PC-3 cells were pre-treated with free HA (5 mg/mL) for 2 h before the incubation and irradiation.

### Targeted ability of ICG@FeDH

Targeted ability of ICG@FeDH was also investigated by CLSM. Typically, PC-3 cells were seeded in culture dish at density of 2 × 10^5^ cells/dish for 24 h. Subsequently, the cells were cultured with ICG@FeD and ICG@FeDH for 2 h. After removing the medium, the cells were washed, fixed by 4% paraformaldehyde and imaged by CLSM under oil lens. Moreover, the cells pre-treated with free HA were also incubated with ICG@FeDH and observed by CLSM. The cells without treatments were set as control.

### In vitro cytotoxicity and cell killing effect

The cytotoxicity of free FeDH was assessed by standard CCK-8 assay. In a typical process, PC-3 cells were seeded into 96-well plates at a density of 1 × 10^4^ cells/well for 24 h. Then the medium was replaced with different concentrations of FeDH, and the cells were further incubated for another 24 h. After that, the cells were rinsed with PBS. After 4 h incubation, the medium was replaced with CCK-8 work solution and incubated at 37 °C for 2 h. The absorbance at 450 nm of each well was measured by a microplate reader. The cell viability was calculated by the value of the control group divided by the values of the samples [36]. Four parallel experiments were set for each sample.

To evaluate the cancer cell killing effect, PC-3 cells were seeded into 96-well plates at a density of 1 × 10^4^ cells/well for 24 h, and further incubated with ICG@FeDH (100 μg/mL) for 4 h. Then the cells were irradiated by 808 nm laser for 10 min, followed by incubated at 37 °C for another 20 h. For comparison, the cells treated with complete medium, NIR laser alone, and ICG@FeDH alone were set as different groups with corresponding procedures. Last, the cell viability was evaluated by CCK-8 assay as described above. The cells were also treated with ICG@FeDH (100 μg/mL) for 4 h, and irradiated by 808 nm laser for different time intervals to evaluate the time-dependent killing effect. Other steps were performed as same as above-mentioned protocols.

### In vivo antitumor effect

All animal experiments were approved by the Animal Care and Use Committee of Southern Medical University, Guangzhou, China. 4–6 weeks-old male nude Balb/c mice were provided by Experimental Animal Center of Southern Medical University. The tumor model was established by subcutaneously injecting PC-3 cells (2 × 10^6^ cells) into right back of the mice. After the tumor size reached ~ 50 mm^3^, the tumor-bearing mice were randomly divided into four groups (n = 4): PBS (control group), NIR laser alone (irradiation group), ICG@FeD with laser irradiation (non-targeted group) and ICG@FeDH with laser irradiation (targeted group). Correspondingly, the experimental mice were intravenously injected with different samples (4 mg/mL, 0.2 mL). For irradiation groups, the tumor site of mice was exposed to 808 nm laser for 10 min. The treatments were performed at day 1 and day 3. After that, the tumor size and body weight of experimental mice were monitored every two days. Moreover, the tumors of each group were extracted for hematoxylin & eosin and immunofluorescent staining.

### Statistical analysis

The results were expressed as mean ± standard deviation. The significance was analyzed by one-way analysis of variance (ANOVA) statistical method and Scheffe's post hoc test. The criteria was set as *p < 0.05 and **p < 0.01 for statistical significance.

## Results and discussion

### Synthesis and characterization of FeDH

**Scheme 1:**
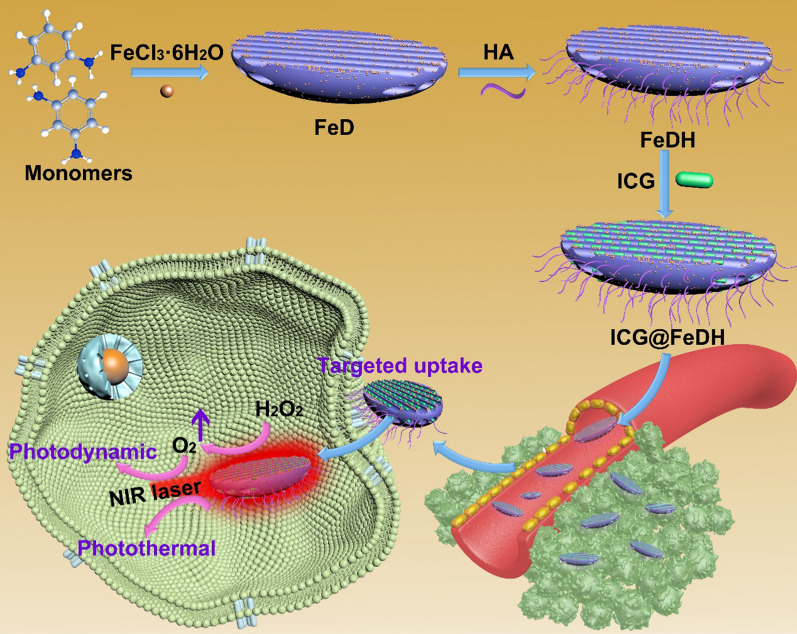
Fabrication of polymer-based nanomedicine for targeted and oxygen-evolved phototherapy of tumor

**Fig. 1:**
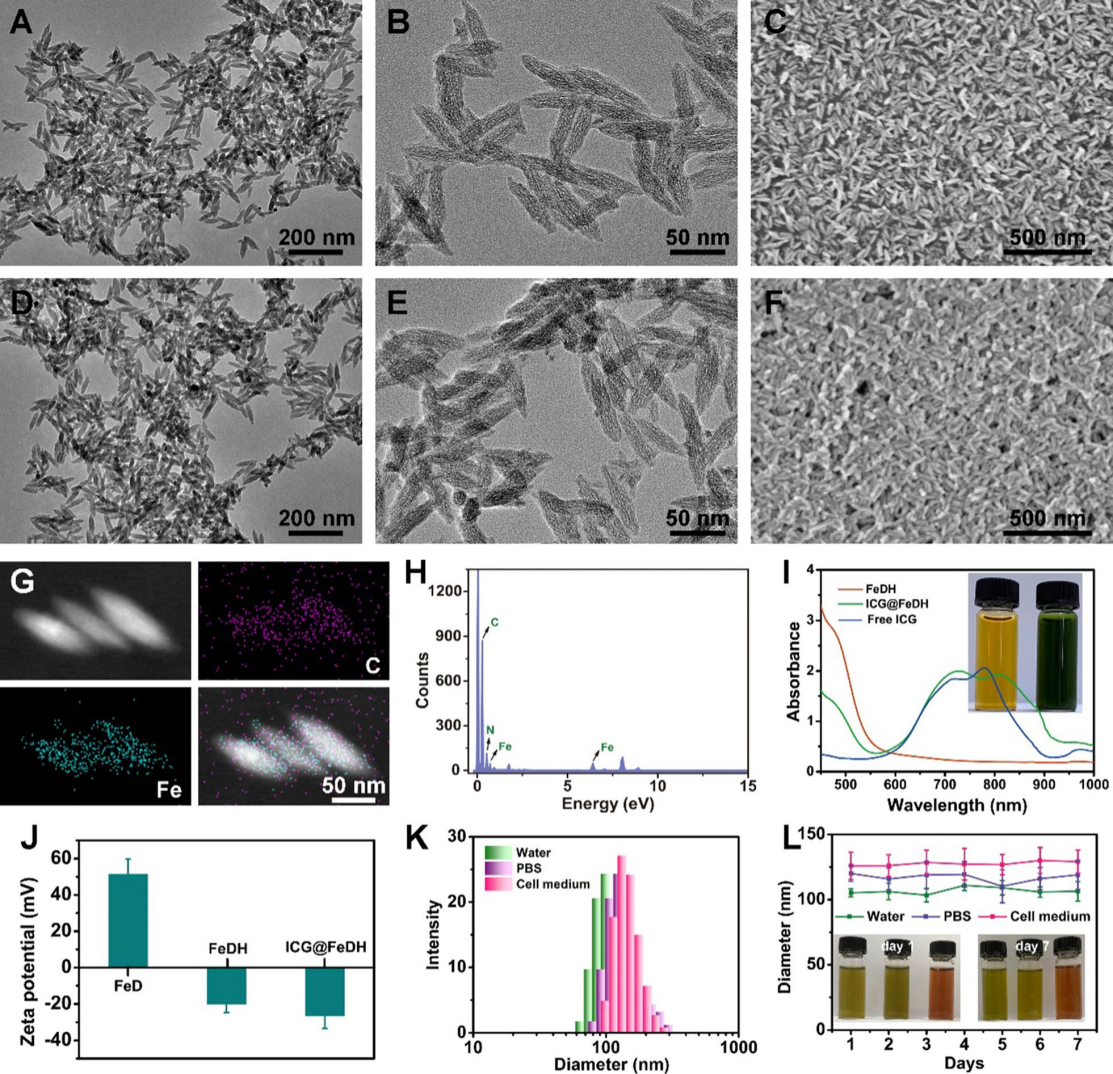
Transmission electron microscopy (TEM) images of **A**, **B** FeD and **D**, **E** FeDH. Scanning electron microscopy (SEM) images of **C** FeD and **F** FeDH. **G** High-angle annular dark field scanning TEM (HAADF-STEM) image and element mapping of FeDH. **H** Energy dispersive X-ray spectrum of FeDH. **I** UV–vis spectra of free ICG, FeDH, and ICG@FeDH. Inset is the photos of FeDH (left) and ICG@FeDH (right) dispersions. **J** Zeta potential results of different samples. **K** Size distributions of FeDH dispersed in different media. **L** Average size of FeDH in different media measured by dynamic light scattering (DLS) within 1 week

As depicted in Scheme 1, the FeD was first prepared by iron ions-initiated polymerization of DAP monomer [37]. TEM images show that as-prepared FeD nanoparticles exhibit fusiform-like structure with a length of 71.4 ± 3.8 nm and width of 16.9 ± 2.1 nm (Fig. 1A). Unlike the spherical nanoparticles, fusiform-like morphology probably endows the nanocarriers with some specific advantages on biological effect. For instance, the mesoporous silica nanorods have been reported with enhanced cell internalization, higher drug loading capacity and improved tumor accumulation in comparison with mesoporous silica nanospheres [38–40]. However, the systematical investigation of the shape effect of covalent organic polymers has rarely been reported, which is mainly due to that the controlled synthesis of covalent organic polymers in different morphology and size still remains a huge challenge to be overcame. The porosity of FeD nanoparticles can be clearly seen in high-magnification TEM image. Moreover, SEM image demonstrate the uniform morphology of FeD in large scale (Fig. 1C). Due to the abundant amino groups on FeD, the targeted molecule HA can be modified onto it by amide reaction. The obtained FeDH maintains the original structure, and the pores are still visible in TEM image (Fig. 1D–F). Further, the element mapping of FeDH is recorded on scanning TEM. Notably, strong signals from iron element can be observed, the energy dispersive X-ray spectrum also confirms the existence of iron ions in FeDH (Fig. 1G, H). It has been reported the iron ions (Fe^3+^) can react with hydrogen peroxide by Fention-like reaction [41, 42], modulating the tumor microenvironment and producing sufficient oxygen to enhance the therapeutic efficacy. Thus, ICG is selected as photosensitizers to be loaded in FeDH. Accordingly, the typical absorption peak of ICG at NIR region for ICG@FeDH verifies the efficient loading process (Fig. 1I and Additional file 2: Figure S1). The free ICG exhibits maximum absorption peaks around 780 nm. It is noted that the characteristic peak of ICG shows a red shift to around 810 nm for ICG@FeDH, which is probably resulted from the interactions between loaded ICG molecules and metal-doped FeDH as well as the self-aggregation of ICG during the loading process [43]. The encapsulation efficiency and loading content is calculated to be 52% and 11.5% on the basis of UV–vis spectrophotometer, respectively. Additionally, the zeta potential of prepared samples is also measured. The bare FeD displays strong positive surface due to the amino groups of DAP (Fig. 1J). Zeta potential of FeDH turns to − 20.3 mV after modification of HA and the ICG@FeDH also exhibits negative surface, which is expected to benefit the colloidal stability of nanoparticles [44]. Then the colloidal stability of FeDH is inspected by DLS. As expected, the size distribution of FeDH in different media, including water, PBS and cell culture medium, shows no abnormality but slight increase in PBS and cell medium (Fig. 1K and Additional file 3: Figure S2). The larger size of FeDH in cell medium can be attributed to the absorption of albumen in cell medium onto its surface as demonstrated by previous report [45]. Moreover, the average size of FeDH exhibits no obvious change in one week, and no agglomeration appears after one week storage (Fig. 1L), suggesting the excellent colloidal stability of FeDH.

### Photothermal effect of ICG@FeDH

**Fig. 2:**
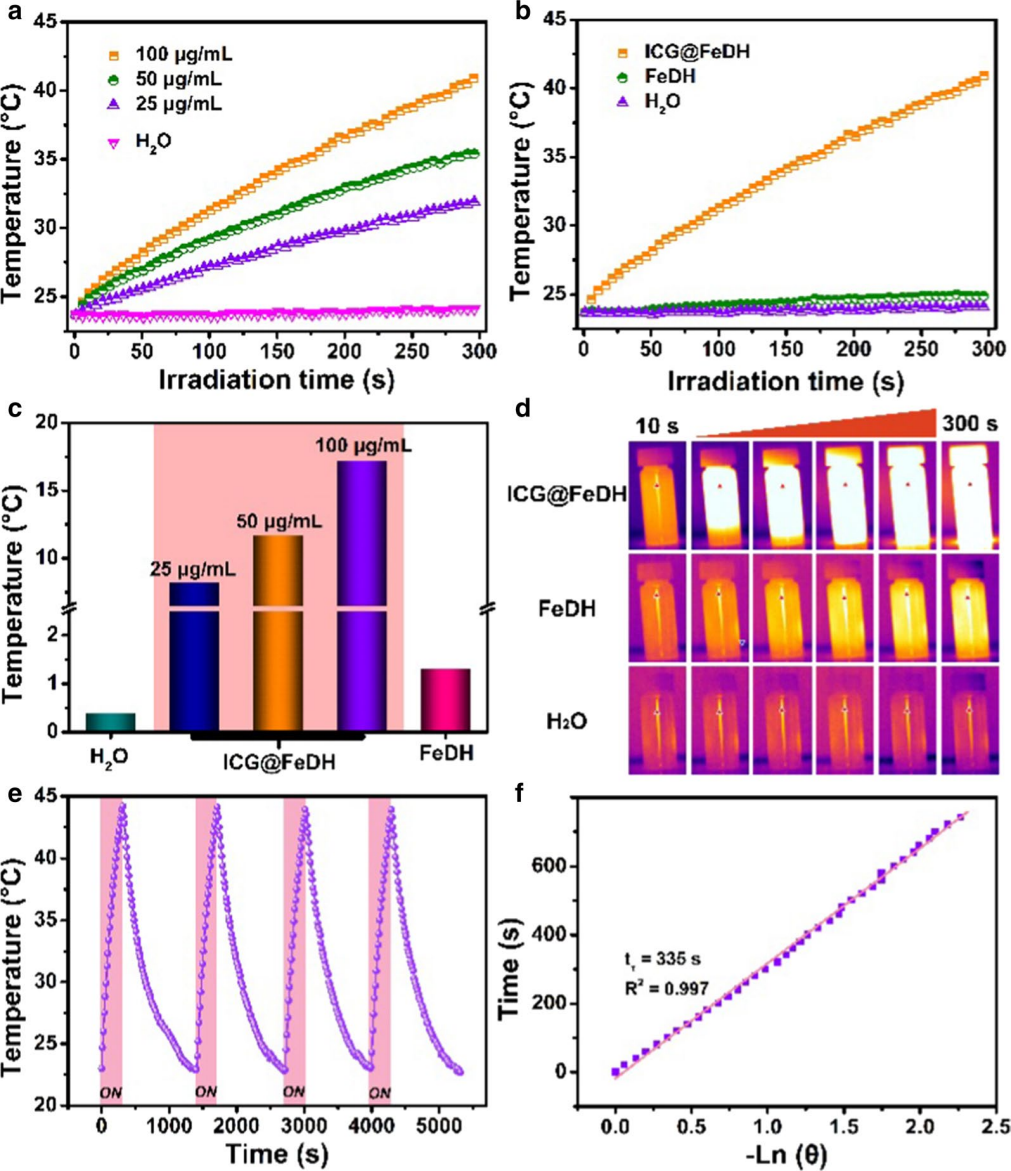
**a** The temperature change versus irradiation time of FeDH aqueous dispersions at different concentrations. Curves. **b** The heating curve of ICG@FeDH, FeDH and H_2_O under the same conditions (1 W/cm^2^, 5 min). **c** Temperature change value and **d** thermal imaging photos at different time points of the corresponded samples in **b**. **e** Temperature change of ICG@FeDH dispersions exposed to photothermal heating and natural cooling cycles under 808 nm laser irradiation. **f** Measurement of photothermal conversion efficiency of ICG@FeDH at 808 nm

Since the ICG@FeDH shows strong NIR absorption, its photothermal property is evaluated next. First, the ICG@FeDH solutions with different concentrations were irradiated with 808 nm laser for 5 min. The temperature of the solutions exhibit an obvious concentration-dependent increase (Fig. 2a), which gradually elevates to 42.3 °C at a low concentration of 100 μg/mL and power density of 1.0 W/cm^−2^. However, the temperature of free FeDH solutions changes 1.2 °C after 5 min of irradiation, and the pure water only 0.3 °C under the same conditions (Fig. 2b, c). The strong contrast of pseudo-color in thermal imaging photos also demonstrates the superior photothermal effect of ICG@FeDH than that of free FeDH (Fig. 2d). Furthermore, the photostability of ICG@FeDH and ICG@FeD is assessed. The dispersions are subjected to three rounds of repeated laser irradiation. Remarkably, the elevation of temperature maintains well without any decrease for both ICG@FeDH and ICG@FeD (Fig. 2e and Additional file 1: Figure S3). The excellent photostability can be attributed to the loading of ICG in FeDH considering the susceptible photobleaching of free ICG [46]. In addition, the photothermal conversion efficiency of ICG@FeDH and ICG@FeD is also calculated to be 19.7% and 19.5%, respectively (Fig. 2f and Additional file 4: Figure S3). The values are apparently lower than previously reported semiconducting polymeric nanoparticles (SPNs) [47, 48], which is due to that the polymerization of small molecules into macromolecules can improve their optical properties [49, 50]. Anyway, the results confirm that the prepared ICG@FeDH can be used as an excellent and stable photothermal agent.

### In vitro photodynamic effect of ICG@FeDH

**Fig. 3:**
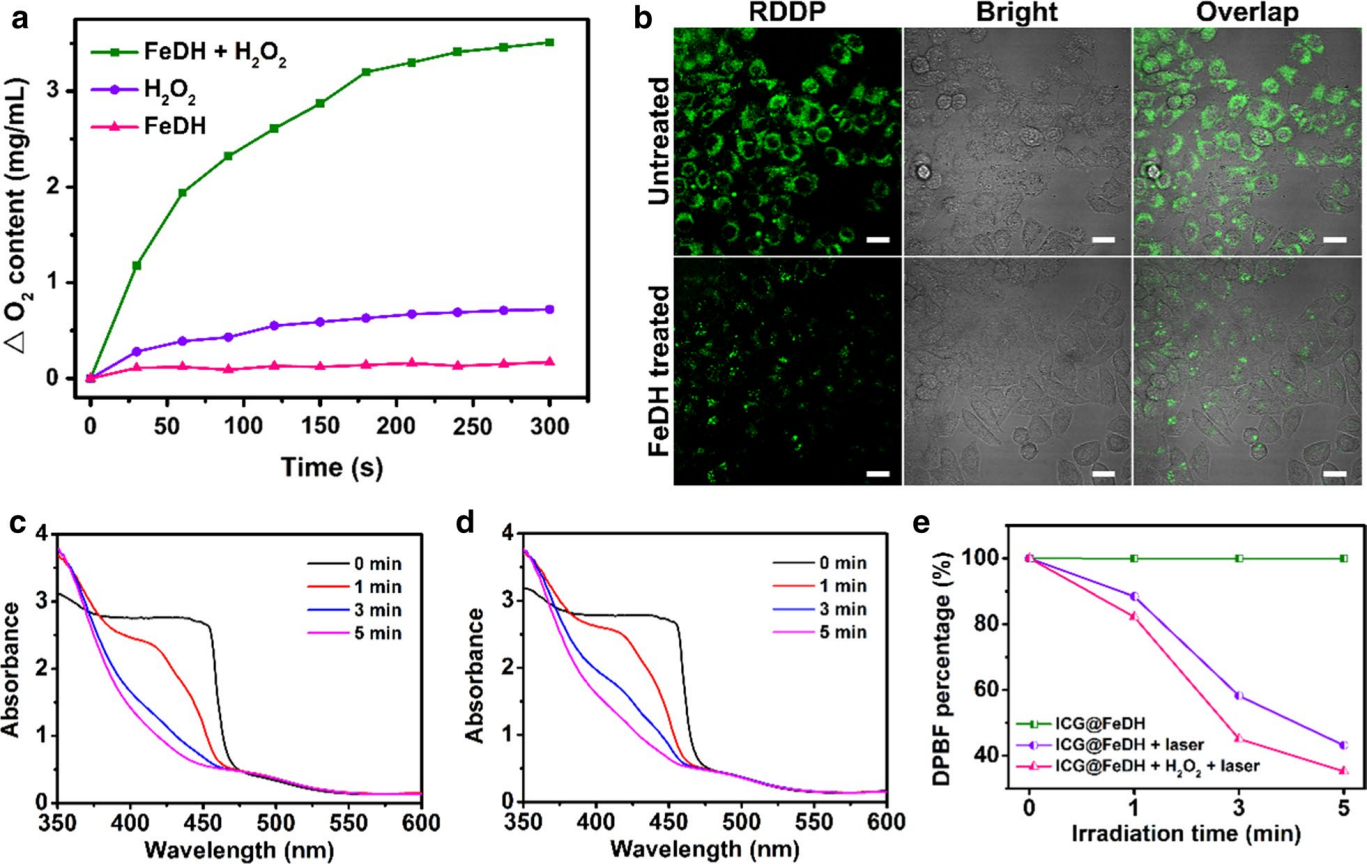
**a** The oxygen content of different samples measured by digital oxygen meter. **b** Confocal laser scanning microscopy (CLSM) images of PC-3 cells after stained with RDDP for 2 h and incubated with or without FeDH for another 2 h. **c** UV–vis spectra of DPBF solutions mixed with (**c**) ICG@FeDH plus hydrogen peroxide and **d** ICG@FeDH under 808 nm laser irradiation for different time points. **e** The calculated degradation rate of DPBF based on the absorbance values at 419 nm in **c**, **d**

In addition to the enhanced photothermal effect, ICG can also generate singlet oxygen under NIR laser irradiation [51]. Thus, the photodynamic effect of ICG@FeDH is investigated. On the other hand, the doped iron ions of FeDH can catalyze the decomposition of hydrogen peroxide. The oxygen level of FeDH solution rapidly increases with the addition of hydrogen peroxide, while the pure water or hydrogen peroxide alone cannot produce oxygen under the same conditions (Fig. 3a). The catalase-like activity of FeDH is further confirmed in living cells by a commercial O_2_ sensing probe RDPP [52]. No surprisingly, the green fluorescence of RDPP in cells treated with FeDH was dramatically weakened as compared to the cells without treatments (Fig. 3b). The result evidences that iron ions doped in FeDH can efficiently catalyze the decomposition of hydrogen peroxide to generate intracellular oxygen, thereby relieving the hypoxia condition in tumor microenvironment. Based on this, the photodynamic effect of ICG@FeDH is also expected to be improved due to the elevated oxygen level. To illustrate this issue, another probe DPBF is used to evaluate the singlet oxygen generation ability of ICG@FeDH under excitation of NIR laser. As expected, the absorption peak of DPBF decreases with the irradiation time of NIR laser (Fig. 3c), implying the generation of singlet oxygen. Moreover, the decrease of the absorption value becomes faster when hydrogen peroxide is added into the ICG@FeDH solution (Fig. 3d). Specifically, after 5 min irradiation, 64.8% of DPBF is oxidized in the presence of hydrogen peroxide (Fig. 3e), which is much high than that of ICG@FeDH alone (57%). Further, the singlet oxygen generation ability of ICG@FeD and ICG@FeDH is further evaluated by SOSG. The fluorescent intensity enhancement (F/F_0_) is calculated according to the previously reported method [53, 54]. The value of F/F_0_ for ICG@FeDH and ICG@FeD is determined to be 3.82 and 3.79 (Additional file 5: Figure S4), respectively, suggesting that the modification of HA would not affect the singlet oxygen generation ability of ICG. Meanwhile, the value increases to 4.57 with the participation of H_2_O_2_ for ICG@FeDH, which is consistent with the results of DPBF. Combined with the decomposition of H_2_O_2_ catalyzed by FeDH, the result can demonstrate that the oxygen-evolving capacity of FeDH can improve the ROS generation of ICG@FeDH under NIR laser irradiation.

### Targeted ability of ICG@FeDH

**Fig. 4:**
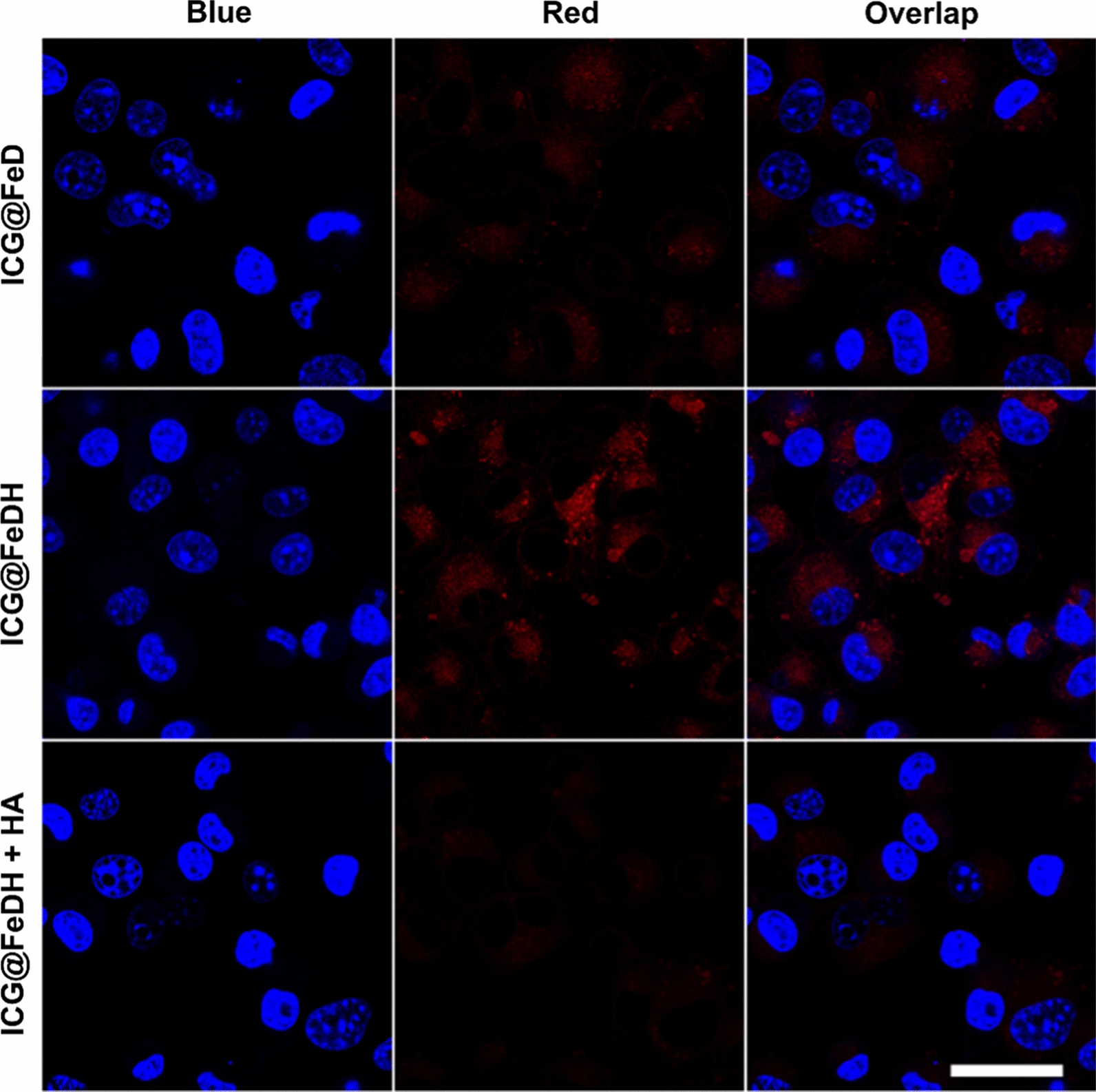
CLSM images of PC-3 cells treated with ICG@FeD, ICG@FeDH and ICG@FeDH with free HA pre-treatment for 2 h. Blue color represents DAPI, red color represents the fluorescence of ICG. Scale bar is 40 μm

With specific affinity towards CD44-receptor [55, 56], the attachment of HA on FeDH can render it with targeted ability towards tumor cells. To demonstrate this, the intracellular uptake of ICG@FeDH is investigated by CLSM using PC-3 cells. After the cells were co-incubated with ICG loaded nanoparticles for 4 h, the red fluorescence of ICG is very weak for non-targeted ICG@FeD. In sharp contrast, strong red fluorescence can be observed around the nucleus for ICG@FeDH group (Fig. 4), which can be attributed to the specific binding of HA with CD44 receptors on PC-3 cells. Once the receptors are inhibited by treating PC-3 cells with free HA, the intracellular fluorescent intensity for ICG@FeDH obviously decreases, further confirming the receptor-mediated endocytosis of ICG@FeDH. Taken together, the modification of HA endows FeDH with excellent targeted ability for tumor therapy.

### In vitro evaluation of ICG@FeDH

**Fig. 5:**
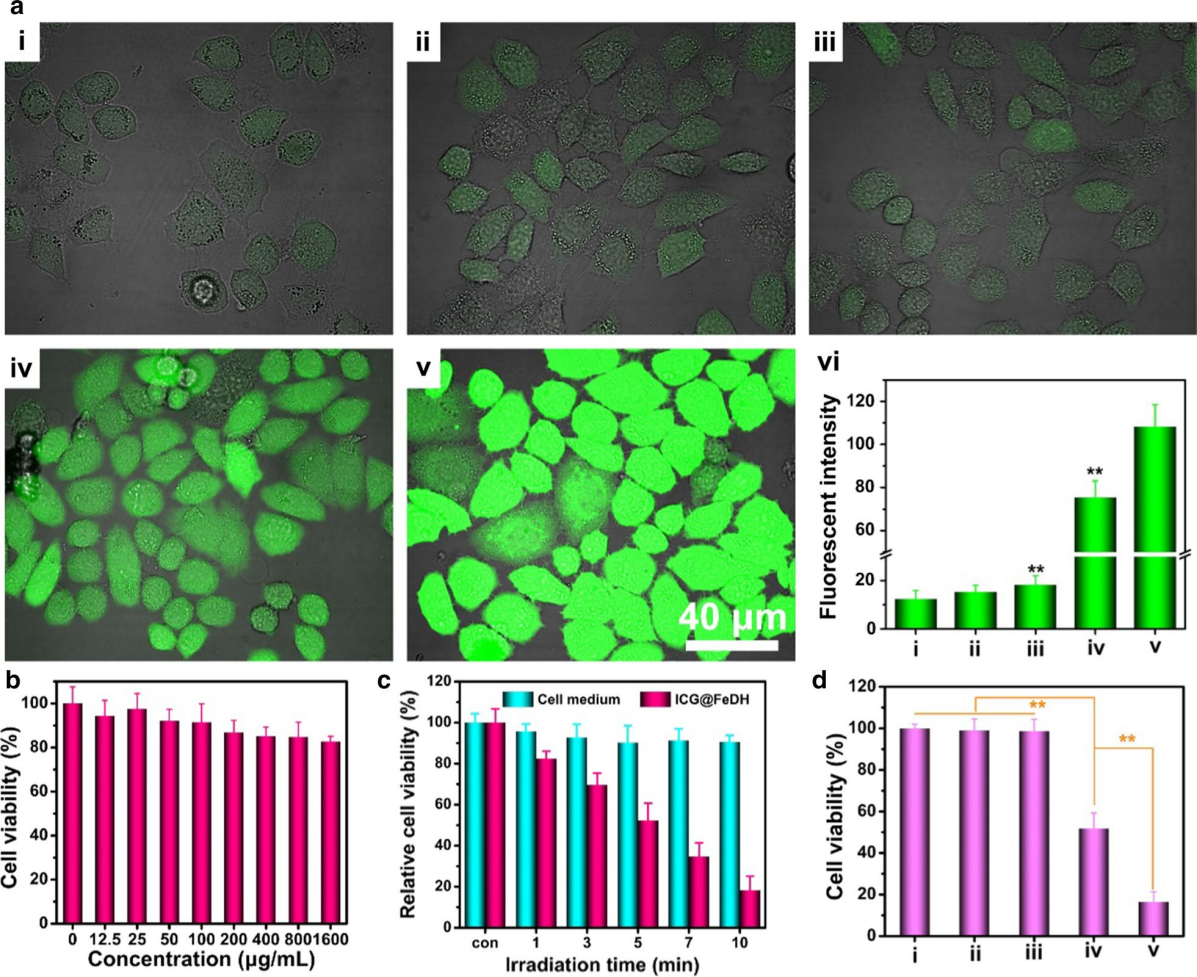
**a** CLSM images of DCFH-DA stained PC-3 cells after treated with (i) complete culture medium, (ii) NIR laser irradiation, (iii) ICG@FeDH alone, (iv) ICG@FeD plus laser irradiation and (v) targeted ICG@FeDH plus laser irradiation. And (vi) The mean fluorescence determined from the corresponded CLSM images. **b** Cell viability of PC-3 cells incubated with FeDH at different concentrations for 24 h. **c** Cell viability of PC-3 cells treated with ICG@FeDH after different irradiation time. **d** Cell viability of PC-3 cells subjected to the corresponding treatments: (i) complete culture medium, (ii) NIR laser irradiation, (iii) ICG@FeDH alone, (iv) ICG@FeD plus laser irradiation and (v) targeted ICG@FeDH plus laser irradiation

Next, the tumor cell killing effect of ICG@FeDH is assessed using PC-3 cells. With the aforementioned photothermal and photodynamic effect, ICG@FeDH is supposed to exert favorable killing effect on tumor cells under NIR laser irradiation. DCFH-DA, a probe molecule that can be oxidized to emit fluorescence at 488 nm [57], is applied to assess the cellular amount of reactive oxygen species (ROS), the major killing mechanism of photodynamic therapy. As shown in Fig. 5a, the cells treated with laser irradiation or ICG@FeDH alone display very weak fluorescence, while the bright green fluorescence can be observed upon laser irradiation for both ICG@FeD and ICG@FeDH treated cells. In particular, the fluorescent intensity of ICG@FeDH with laser irradiation is determined to be 108.3 (Fig. 5b), which is much stronger than that of ICG@FeH with laser irradiation (~ 75.4), demonstrating the higher ROS level for ICG@FeDH. The result can be ascribed to the enhanced cellular uptake of targeted ICG@FeDH as mentioned above.

To further evaluate the cell killing efficacy, the cytotoxicity of free FeDH is investigated first. The result of CCK-8 demonstrates that the cells treated with various concentrations of FeDH all show negligible toxicity even at a ultra-high concentration of 1600 μg/mL, suggesting the outstanding biocompatibility of FeDH. Thus, this polymer-based nanocarrier is highly suitable for biological applications in comparison to those non-degradable inorganic nanomaterials [58]. Afterwards, the killing effect is assessed using PC-3 cells. The time-dependent mortality can be observed upon the tumor cells subjected to ICG@FeDH plus NIR laser irradiation (Fig. 5c), demonstrating the outstanding phototherapeutic efficacy of ICG@FeDH. Moreover, cells treated with ICG@FeDH or laser irradiation alone exhibit high cell viability over 95% (Fig. 5d), suggesting the minimal damage of NIR laser alone. The superior cell killing efficacy of ICG@FeDH is probably due to its excellent photothermal and photodynamic effect under laser irradiation. Combining with the modification of HA, the ICG@FeDH can serve as a targeted phototherapeutic agent for killing tumor cells.

### In vivo antitumor effect of ICG@FeDH

**Fig. 6:**
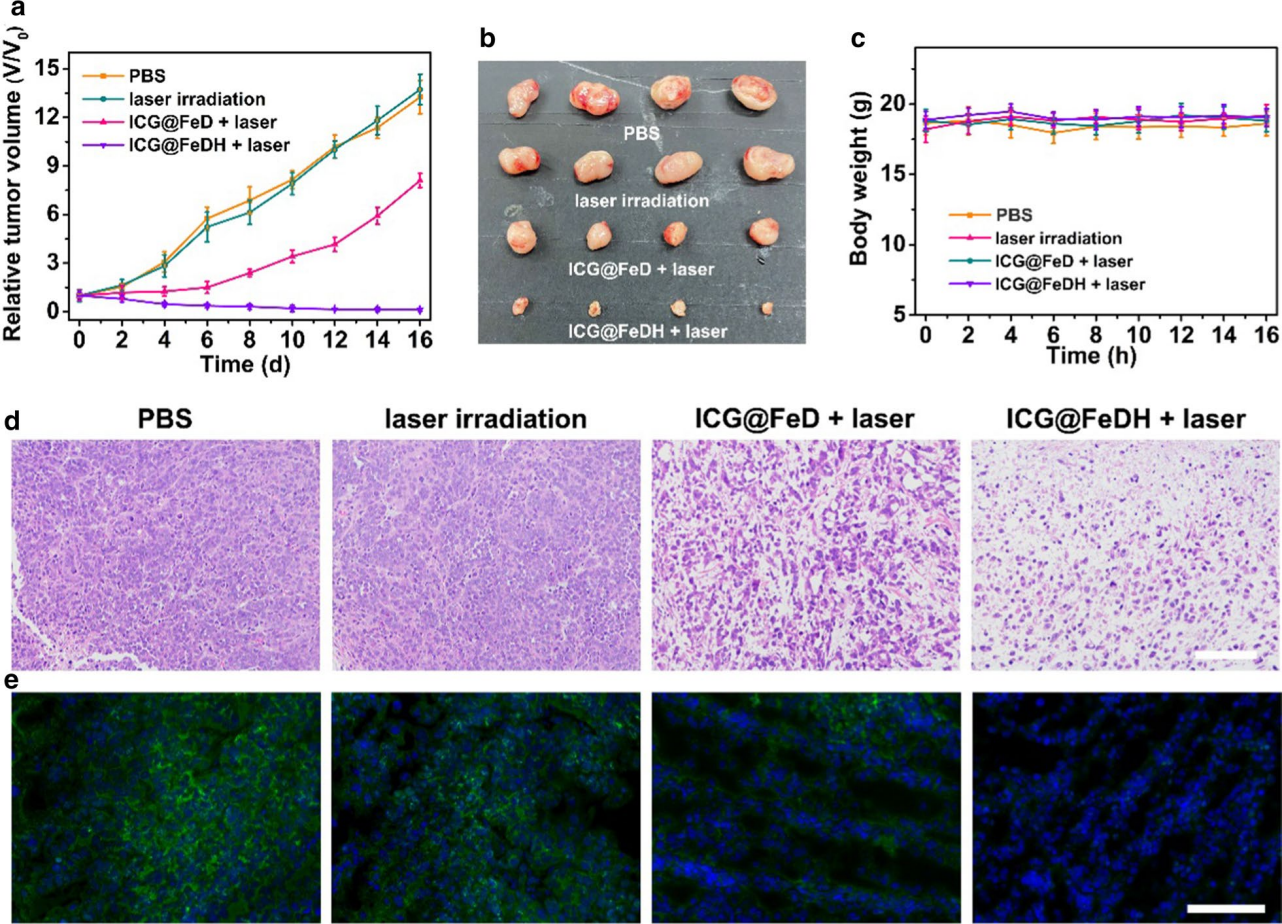
**a** The change of relative tumor volume within two weeks under different treatments. **b** The tumor photographs extracted from mice of different groups at the end of treatments. **c** The change of body weights of experimental mice of different groups in the period of treatments. **d** H&E staining and **e** Immunofluorescence staining for HIF-1α expression level of histological sections from tumor tissues in different groups. Green color in **d** represents HIF-1α and Blue color represents DAPI. Scale bar in **c**, **d** is 200 μm

Next, the in vivo therapeutic efficacy of ICG@FeDH was investigated on PC-3 tumor-bearing mice. To implement the treatments, the mice are intravenously injected with ICG@FeDH and NIR laser irradiation is conducted. Figure 6a shows the variation of relative tumor volume in the period of treatment. The tumors treated by ICG@FeDH with 808 nm laser irradiation were remarkably inhibited and displayed a relative tumor volume of 0.38 on day 6 without any recurrence, implying the superior phototherapeutic efficacy of ICG@FeDH under NIR laser irradiation. As for control groups of PBS and NIR laser alone, the size of tumors are rapidly increased within 2 weeks (Fig. 6b), showing negligible therapeutic effect. Notably, the ICG@FeD with laser irradiation shows an inferior tumor inhibition rate in comparison to the counterpart of ICG@FeDH, which confirms that the modification of HA can improve its in vivo antitumor effect. Besides, the body weight of experimental mice shows no obvious change in the period of treatments (Fig. 6c), indicating that the well-tolerance and excellent biocompatibility of the applied samples. Furthermore, the tumors are harvested at the end of treatments and stained with H&E for histological analysis. The images show that no apparent necrosis appears in control group (Fig. 6d). Moreover, the group of ICG@FeDH plus laser irradiation exhibits much more necrosis and karyolysis in slice than that in the group of non-targeted ICG@FeD plus laser irradiation. It is deduced that the better therapeutic efficacy of ICG@FeDH is mainly resulted from targeted ability of HA as well as the improved photodynamic effect because of the oxygen-evolved capacity of FeDH. To better understand this, the immunofluorescent staining of hypoxia-inducible factor 1α (HIF1-α) is conducted to detect the oxygen level in tumor. The green fluorescence indicative of hypoxia remarkably reduces in the group of ICG@FeDH (Fig. 6e and Additional file 6: Figure S5). It should be noticed that the fluorescence in the group of ICG@FeD is also weakened as compared with control group and NIR laser group, but still stronger than ICG@FeDH, which is probably due to that the efficient targeted ability of ICG@FeDH render it with higher accumulation at tumor site. Taken together, ICG@FeDH effectively integrates several advantages, including excellent biocompatibility and targeted ability as well as oxygen-evolved capacity for enhanced phototherapeutic efficacy, showing great potential for biomedical applications.

## Conclusion

In summary, a new type of polymer-based nanocarrier is fabricated by oxidize polymerization for efficiently targeted and oxygen-evolved phototherapy of tumor. The synthesized FeD shows fusiform-like structure with average size of ~ 70 nm. With the surface modification of HA, the obtained FeDH not only possesses outstanding colloidal stability and excellent biocompatibility for biomedical application. More importantly, the doped iron ions in FeDH are demonstrated with catalase-like activity, which can catalyze the generation of oxygen in hydrogen peroxide-excessive tumor microenvironment. After loading the photosensitizer ICG into FeDH, the obtained FeDH demonstrates favorable photothermal effect, enhanced photodynamic effect and specific binding affinity towards PC-3 tumor cells, achieving efficiently targeted and combined phototherapy of tumor in vitro and in vivo. Overall, this simple but efficient nanoagent provides a new paradigm of polymer-based nanocarrier for enhanced phototherapeutic efficacy.

## Supplementary information

**Additional file 1.** The calculation method of photothermal conversion efficiency of ICG@FeD and ICG@FeDH.

**Additional file 2: Figure S1.** The photographs of ICG@FeDH dispersion (left) before and (right) after high-speed centrifugation. The loaded ICG is precipitated together with FeDH and no obvious green color can be observed in supernatant solution, indicating the efficient loading of ICG.

**Additional file 3: Figure S2.** Size distribution of ICG@FeDH measured by DLS. The size distribution of ICG@FeDH shows no abnormal change in comparison to bare FeDH, implying that the loading of ICG would not affect the colloidal stability of FeDH.

**Additional file 4: Figure S3.** (A) Temperature change of ICG@FeD dispersions exposed to photothermal heating and natural cooling cycles under 808 nm laser irradiation. (B) Measurement of photothermal conversion efficiency of ICG@FeD at 808 nm.

**Additional file 5: Figure S4.** The fluorescence intensity changes of SOSG at 525 nm withincreasing irradiation time.

**Additional file 6: Figure S5.** Quantification of HIF-α expression in tumor slices from different groups. **P<0.01..
