# Supplementary material for: Hyaluronic acid modified covalent organic polymers for efficient targeted and oxygen-evolved phototherapy
Source: J Nanobiotechnology. 2021 Jan 6;19:4. doi: 10.1186/s12951-020-00735-x (PMC7789517; doi:10.1186/s12951-020-00735-x)
Supplement: Supplementary file 1 — Additional file 1. The calculation method of photothermal conversion efficiency of ICG@FeD and ICG@FeDH. [file 12951_2020_735_MOESM1_ESM.docx]

**Hyaluronic acid modified covalent organic polymers for efficient targeted and oxygen-evolved phototherapy**

Fangpeng Shu^1,2#^, Taowei Yang^2#^, Xuefeng Zhang^3^, Wenbin Chen^2^, Kaihui Wu^2^, Junqi Luo^2^, Xumin Zhou^2^, Guochang Liu^3^*, Jianming Lu^2^*, Xiangming Mao^2^*.

^1^ Department of Urology, Guangzhou Women and Children's Medical Center, Guangzhou Medical University, Guangzhou, China.

^2^ Department of Urology, Zhujiang Hospital of Southern Medical University, Guangzhou, China.

^3^ Department of Urology, First Affiliated Hospital of Soochow University, 899 Pinghai Road, Suzhou, 215031, China

^#^ These authors contribute equally to this work.

Corresponding author: Prof. Xiangming Mao ([mxm631221@126.com](mailto:mxm631221@126.com))

Prof. Jianming Lu ([Louiscfc8@gmail.com](mailto:Louiscfc8@gmail.com))

Prof. Guochang Liu ([starbless2003@126.com](mailto:starbless2003@126.com))

***Calculation of photothermal conversion efficiency***

The photothermal conversion efficiency is calculated according to previously reported method^1,2^:

$\sum_{i} m_{i}C_{i}\frac{dT}{dt}=Q_{NC}+Q_{sys}-Q_{out}$ (1)

Where *m* and C are the mass and heat capacity of water, respectively, T is the solution temperature, *Q_NC_* is the energy absorbed by ICG@FeDH and ICG@FeD, *Q_sys_* is the energy imputed by the pure water system, and *Q_out_* is heat dissipation of the system.

The heat absorbed (*Q_NC_*) by ICG@FeDH and ICG@FeD can be shown as:

$Q_{NC}=I(1-10^{-A_{808}})\eta$ (2)

Where *I* is incident laser power in W, $\eta$is the photothermal conversion efficiency, and A_808_ indicates the absorbance of the ICG@FeDH and ICG@FeD at 808 nm.

*Q_out_* is linear with system temperature, as expressed as:

$Q_{out}=hS(T-T_{surr})$ (3)

Where *h* is heat transfer coefficient, *S* is the surface area of the container, and *T_surr_* is ambient temperature of the surroundings.

When the system reaches a steady state temperature (*T_max_*), the heat input and output are balanced:

$Q_{NC}+Q_{sys}=Q_{out}=hS(T_{max}-T_{suur})$ (4)

After the laser is removed, the *Q_NC_* + *Q_sys_* = 0, reducing the Eq. (1)

$\sum_{i} m_{i}C_{i}\frac{dT}{dt}=-Q_{out}=-hS(T-T_{surr})$ (5)

Rearranging the Eq. (5) would give

$dt=-\frac{\sum_{i} m_{i}C_{i}}{hS}\frac{dT}{(T-T_{surr})}$ (6)

And integrating, give the expression

$t=-\frac{\sum_{i} m_{i}C_{i}}{hS}\ln\frac{T-T_{surr}}{(T_{max}-T_{suur})}$ (7)

A system time constant *τ_s_* is defined as:

$\tau_{s}=-\frac{\sum_{i} m_{i}C_{i}}{hS}$ (8)

And *θ* is introduced using the maximum system temperature, *T_max_*

$\theta=\frac{T-T_{surr}}{(T_{max}-T_{suur})}$ (9)

Substituting Eq. (8) and (9) giving:

$t=-\tau_{s}\ln\theta$ (10)

Therefore, the time constant for heat transfer from the system *τ_s_* can be determined by applying the linear time data from the cooling period vs. negative natural logarithm of driving force temperature (*θ*)

Since *Q_sys_* can be obtained directly as

$Q_{sys}=hS(T_{max, H_{2}O}-T_{surr})$ (11)

Eq. (4) can be given as:

$Q_{NC}=I(1-10^{-A_{808}})\eta=hS(T_{max}-T_{max, H_{2}O})$ (12)

Also

$hS=-\frac{\sum_{i} m_{i}C_{i}}{\tau_{s}}$ (13)

Finally, for ICG@FeDH, the *τ_s_* is determined to be 335.17 s. m is 0.5 g and C is 4.2 J/g, hS can be calculated to be -0.006265 W/^o^C. Substituting *I* = 1.0 W/cm^2^, *A_808_* = 0.483, respectively, and *T_max_* - *T_surr_* = 44.2 - 23=21.2 ^o^C into Eq. (12), the photothermal conversion efficiency can be determined to be 19.7%.

Finally, for ICG@FeDH, the *τ_s_* of ICG@FeDH is determined to be 335.42 s. m is 0.5 g and C is 4.2 J/g, hS can be calculated to be -0.006261 W/^o^C. Substituting *I* = 1.0 W/cm^2^, *A_808_* = 0.476, and *T_max_* - *T_surr_* = 43.2 – 22.5=20.7 ^o^C into Eq. (12), the photothermal conversion efficiency can be determined to be 19.5%.

**References**

[1] D. K. Roper, W. Ahn and M. Hoepfner, *J. Phys. Chem. C*, 2007, **111**, 3636-3641.

[2] X. W. Wang, X. Y. Zhong, H. L. Lei, Y. H. Geng, Q. Zhao, F. Gong, Z. J. Yang, Z. L. Dong, Z. Liu and L. Cheng, *Chem. Mater.* 2019, **31**, 6174-6186.
