# Supplementary material for: Hyaluronic acid modified covalent organic polymers for efficient targeted and oxygen-evolved phototherapy
Source: J Nanobiotechnology. 2021 Jan 6;19:4. doi: 10.1186/s12951-020-00735-x (PMC7789517; doi:10.1186/s12951-020-00735-x)
Supplement: Supplementary file 2 — Additional file 2: Figure S1. The photographs of ICG@FeDH dispersion (left) before and (right) after high-speed centrifugation. The loaded ICG is precipitated together with FeDH and no obvious green color can be observed in supernatant solution, indicating the efficient loading of ICG. [file 12951_2020_735_MOESM2_ESM.docx]

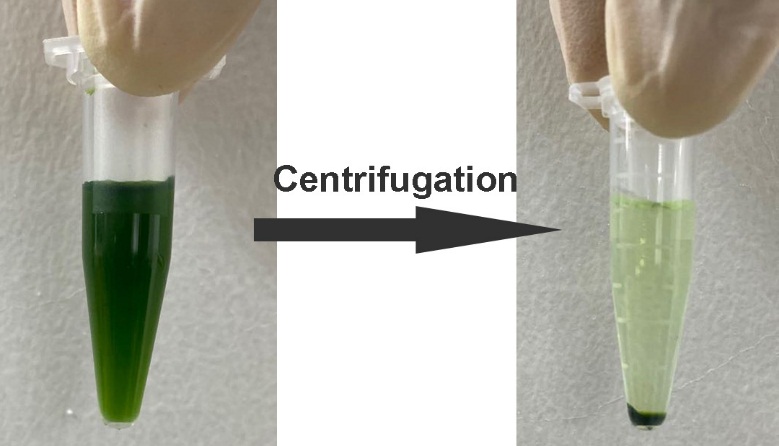


**Figure S1.** The photographs of ICG@FeDH dispersion (left) before and (right) after high-speed centrifugation. The loaded ICG is precipitated together with FeDH and no obvious green color can be observed in supernatant solution, indicating the efficient loading of ICG.
