# Supplementary material for: Hyaluronic acid modified covalent organic polymers for efficient targeted and oxygen-evolved phototherapy
Source: J Nanobiotechnology. 2021 Jan 6;19:4. doi: 10.1186/s12951-020-00735-x (PMC7789517; doi:10.1186/s12951-020-00735-x)
Supplement: Supplementary file 3 — Additional file 3: Figure S2. Size distribution of ICG@FeDH measured by DLS. The size distribution of ICG@FeDH shows no abnormal change in comparison to bare FeDH, implying that the loading of ICG would not affect the colloidal stability of FeDH. [file 12951_2020_735_MOESM3_ESM.docx]

**Figure S2.** Size distribution of ICG@FeDH measured by DLS. The size distribution of ICG@FeDH shows no abnormal change in comparison to bare FeDH, implying that the loading of ICG would not affect the colloidal stability of FeDH.
