# Supplementary material for: Hyaluronic acid modified covalent organic polymers for efficient targeted and oxygen-evolved phototherapy
Source: J Nanobiotechnology. 2021 Jan 6;19:4. doi: 10.1186/s12951-020-00735-x (PMC7789517; doi:10.1186/s12951-020-00735-x)
Supplement: Supplementary file 4 — Additional file 4: Figure S3. (A) Temperature change of ICG@FeD dispersions exposed to photothermal heating and natural cooling cycles under 808 nm laser irradiation. (B) Measurement of photothermal conversion efficiency of ICG@FeD at 808 nm. [file 12951_2020_735_MOESM4_ESM.docx]

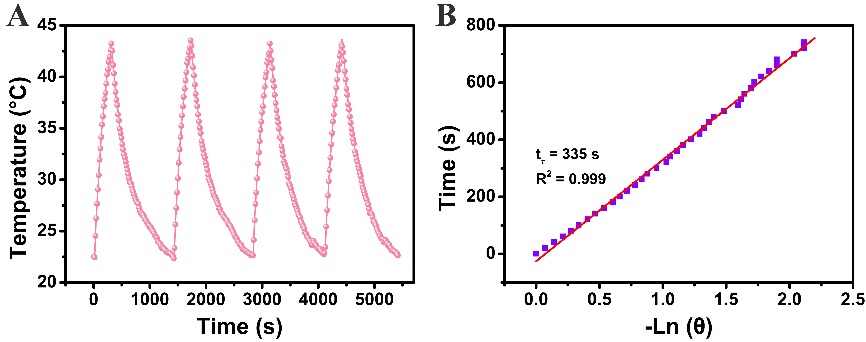


**Figure S3.** (A) Temperature change of ICG@FeD dispersions exposed to photothermal heating and natural cooling cycles under 808 nm laser irradiation. (B) Measurement of photothermal conversion efficiency of ICG@FeD at 808 nm.
