# Supplementary material for: Hyaluronic acid modified covalent organic polymers for efficient targeted and oxygen-evolved phototherapy
Source: J Nanobiotechnology. 2021 Jan 6;19:4. doi: 10.1186/s12951-020-00735-x (PMC7789517; doi:10.1186/s12951-020-00735-x)
Supplement: Supplementary file 5 — Additional file 5: Figure S4. The fluorescence intensity changes of SOSG at 525 nm withincreasing irradiation time. [file 12951_2020_735_MOESM5_ESM.docx]

**Figure S4.** The fluorescence intensity changes of SOSG at 525 nm with increasing irradiation time.
