# Supplementary material for: Hyaluronic acid modified covalent organic polymers for efficient targeted and oxygen-evolved phototherapy
Source: J Nanobiotechnology. 2021 Jan 6;19:4. doi: 10.1186/s12951-020-00735-x (PMC7789517; doi:10.1186/s12951-020-00735-x)
Supplement: Supplementary file 6 — Additional file 6: Figure S5. Quantification of HIF-α expression in tumor slices from different groups. **P<0.01.. [file 12951_2020_735_MOESM6_ESM.docx]

**Figure S5.** Quantification of HIF-α expression in tumor slices from different groups. **P<0.01.
